# Whole genome re-sequencing reveals genome-wide variations among parental lines of 16 mapping populations in chickpea (*Cicer arietinum* L.)

**DOI:** 10.1186/s12870-015-0690-3

**Published:** 2016-01-27

**Authors:** Mahendar Thudi, Aamir W. Khan, Vinay Kumar, Pooran M. Gaur, Krishnamohan Katta, Vanika Garg, Manish Roorkiwal, Srinivasan Samineni, Rajeev K. Varshney

**Affiliations:** International Crops Research Institute for the Semi-Arid Tropics (ICRISAT), Hyderabad, India; The University of Western Australia (UWA), Crawley, Western Australia Australia

**Keywords:** Chickpea, Re-sequencing, Copy number variations, Mapping populations

## Abstract

**Background:**

Chickpea (*Cicer arietinum* L.) is the second most important grain legume cultivated by resource poor farmers in South Asia and Sub-Saharan Africa. In order to harness the untapped genetic potential available for chickpea improvement, we re-sequenced 35 chickpea genotypes representing parental lines of 16 mapping populations segregating for abiotic (drought, heat, salinity), biotic stresses (*Fusarium* wilt, *Ascochyta* blight, *Botrytis* grey mould, *Helicoverpa armigera*) and nutritionally important (protein content) traits using whole genome re-sequencing approach.

**Results:**

A total of 192.19 Gb data, generated on 35 genotypes of chickpea, comprising 973.13 million reads, with an average sequencing depth of ~10 X for each line. On an average 92.18 % reads from each genotype were aligned to the chickpea reference genome with 82.17 % coverage. A total of 2,058,566 unique single nucleotide polymorphisms (SNPs) and 292,588 *Indels* were detected while comparing with the reference chickpea genome. Highest number of SNPs were identified on the Ca4 pseudomolecule. In addition, copy number variations (CNVs) such as gene deletions and duplications were identified across the chickpea parental genotypes, which were minimum in PI 489777 (1 gene deletion) and maximum in JG 74 (1,497). A total of 164,856 line specific variations (144,888 SNPs and 19,968 *Indels*) with the highest percentage were identified in coding regions in ICC 1496 (21 %) followed by ICCV 97105 (12 %). Of 539 miscellaneous variations, 339, 138 and 62 were inter-chromosomal variations (CTX), intra-chromosomal variations (ITX) and inversions (INV) respectively.

**Conclusion:**

Genome-wide SNPs, *Indels*, CNVs, PAVs, and miscellaneous variations identified in different mapping populations are a valuable resource in genetic research and helpful in locating genes/genomic segments responsible for economically important traits. Further, the genome-wide variations identified in the present study can be used for developing high density SNP arrays for genetics and breeding applications.

**Electronic supplementary material:**

The online version of this article (doi:10.1186/s12870-015-0690-3) contains supplementary material, which is available to authorized users.

## Background

Chickpea (*Cicer arietinum* L.) is the second most important grain legume cultivated mostly on residual soil moisture in the arid and semi-arid regions of the world. It is a self-pollinated crop and cross pollination is a rare event (0–1 %) [1]. Chickpea has its origin in south-eastern Turkey, and after its domestication, from a closely related wild species *C. reticulatum* Ladizinsky, in the Middle East this crop progressed further throughout the Mediterranean region, India and Ethiopia [2, 3]. It is a rich source of protein to vegetarian diets, especially in India. Globally, it is cultivated on over 13.5 Mha with an annual production of 13.1 million tons [4] and productivity is less than 1 t/ha which is very much less than estimated potential of 6 t/ha under optimum growing conditions. In India, it is cultivated on 9.6 Mha with 8.8 million tons production and an average productivity of 920 kg/ha. About 71 % of the global area with 67 % of global production of chickpea is contributed by India. Despite being the largest producer, India imports chickpea from several countries e.g. Australia, Turkey, Mexico, USA, Canada etc.

Several biotic and abiotic stress have been affecting the chickpea productivity. However, efforts to increase the productivity could not yield much success due to low genetic diversity in cultivated gene pool [5]. This limited genetic diversity in the cultivate gene pool affects genetics and genomic studies in chickpea as number of markers found polymorphic between parents were comparatively very low in comparison to other crops. For instance after screening more than two thousand markers on intra-specific chickpea populations (ICC 4958 × ICC 1882 and ICC 283 × ICC 8261) only couple of hundred representing ~10 % of total polymorphic markers could be identified for these populations [6]. In cases with low genetic diversity, identification of polymorphic markers between contrasting parents is time consuming and tedious task [7].

Recent advances in next-generation sequencing (NGS) technologies dramatically reduced the cost on sequencing and are being deployed to understand the genome architecture, variations in genomes, identify the candidate genes for biotic and abiotic stresses that limit crop productivity below the production potential [8]. In order to harness the untapped genetic potential available for crop improvement in a species, several germplasm lines have been re-sequenced using whole genome re-sequencing (WGRS) approach in different crops. For instance, 3000 rice genomes [9], maize [10], sorghum [11] etc., have been re-sequenced.

During recent years, small variations in the form of single nucleotide polymorphisms (SNPs) and *Indels* are being extensively deployed in crop improvement. Sometimes these small variations do not capture all the genomic information associated with a particular phenotypic variation. This may be due to other important class of large genomic variations i.e. structural variations (SVs). These SVs include inversions, translocations, transversions, copy number variations (CNVs), insertions and deletions, are genomic rearrangements ranging from 50 nucleotides to several megabases with respect to the reference genome [12, 13]. In case of humans, these large variations are extensively studied and are associated with important complex disease phenotypes. Nevertheless, in case of plants, very few studies explored the usefulness of large variations for instance in maize, SVs have been studied between maize and its progenitor [14], while functional impact and origin mechanisms of CNVs were reported in case of barley [15].

Nevertheless, the availability of draft genome sequence for several crop plants [16] including chickpea [17], opened new vistas for crop improvement strategies. Understanding genome wide variation among parental lines of mapping populations will enable trait mapping and identification of stress responsive candidate genes. With an objective to understand the genome-wide variations that can be deployed for chickpea improvement, we re-sequenced a set 35 genotypes that are parental lines of 16 mapping populations and segregate for different biotic and abiotic stresses as well as nutritionally important traits in chickpea.

## Results and discussion

To dissect complex biotic and abiotic stresses, several bi-parental mapping populations and next generation mapping populations like multi-parent advanced generation intercross (MAGIC) population are being used at ICRISAT. Although few thousand simple sequence repeat (SSR) markers are available for trait mapping in chickpea, limited polymorphism among parental lines of bi-parental mapping population has been hindering the trait mapping efforts to reach to the candidate genes responsible for the traits of interest [7]. Nevertheless, genome-wide variations like SNPs, CNVs and PAVs are very important for trait mapping and crop improvement and gaining importance in recent years.

In order to gain insights into the genome-wide variations that can be used for trait dissection and in-turn for chickpea improvement, 35 chickpea genotypes with diverse origin (India, Mexico, Turkey, Tanzania, Commonwealth of Independent States and Russia) and representing both market classes (desi and kabuli) were re-sequenced in this study. These 35 chickpea genotypes are parental lines of 16 mapping populations segregating for abiotic (drought, heat, salinity), biotic stresses (*Fusarium* wilt, *Ascochyta* blight, *Botrytis* grey mould, *Helicoverpa* pod borer) and nutritionally important (protein content) traits; parental lines of MAGIC population and parental lines of marker-assisted recurrent selecion (MARS) populations (Additional file 1).

### *In silico* mapping of sequence data 

A total of 192.19 Gb comprising of 973.13 million 150 and 100 bp reads were generated for 35 genotypes of chickpea at an average sequencing depth of 10.32X for each line (Additional file 2). The trimming and processing of the data resulted in 911.22 million high quality reads. On aligning the clean data, using Bowtie 2, to the CDC Frontier reference genome the mapping rate of reads varied from 90.19 % (IG 72953) to 95.3 % (JG 62). The variation in mapping rate among different genotypes may be due to divergence among the parental genotypes used in the study. On an average 92.18 % reads from each genotype were aligned to the reference genome with 82.17 % average coverage. The number of reads from each genotype aligned on to reference genome varied from 12,765,493 (ICC 1496) to 87,487,094 (JAKI 9218) while uniquely aligned reads varied from 7,778,952 (ICC 1496) to 40,072,407 (JAKI 9218) thus on an average 53.92 % high quality reads were uniquely aligned to the genome. The mean depth ranged from 5.79 to 20.04 with an average of ~8.6 for all the samples. Higher mean depths of 14.26 and 20.04 were observed in ICC 4958 and JAKI 9218 because of comparatively higher amount of reads generated for these samples (Additional file 2).

### SNPs and their distribution

**Fig. 1:**
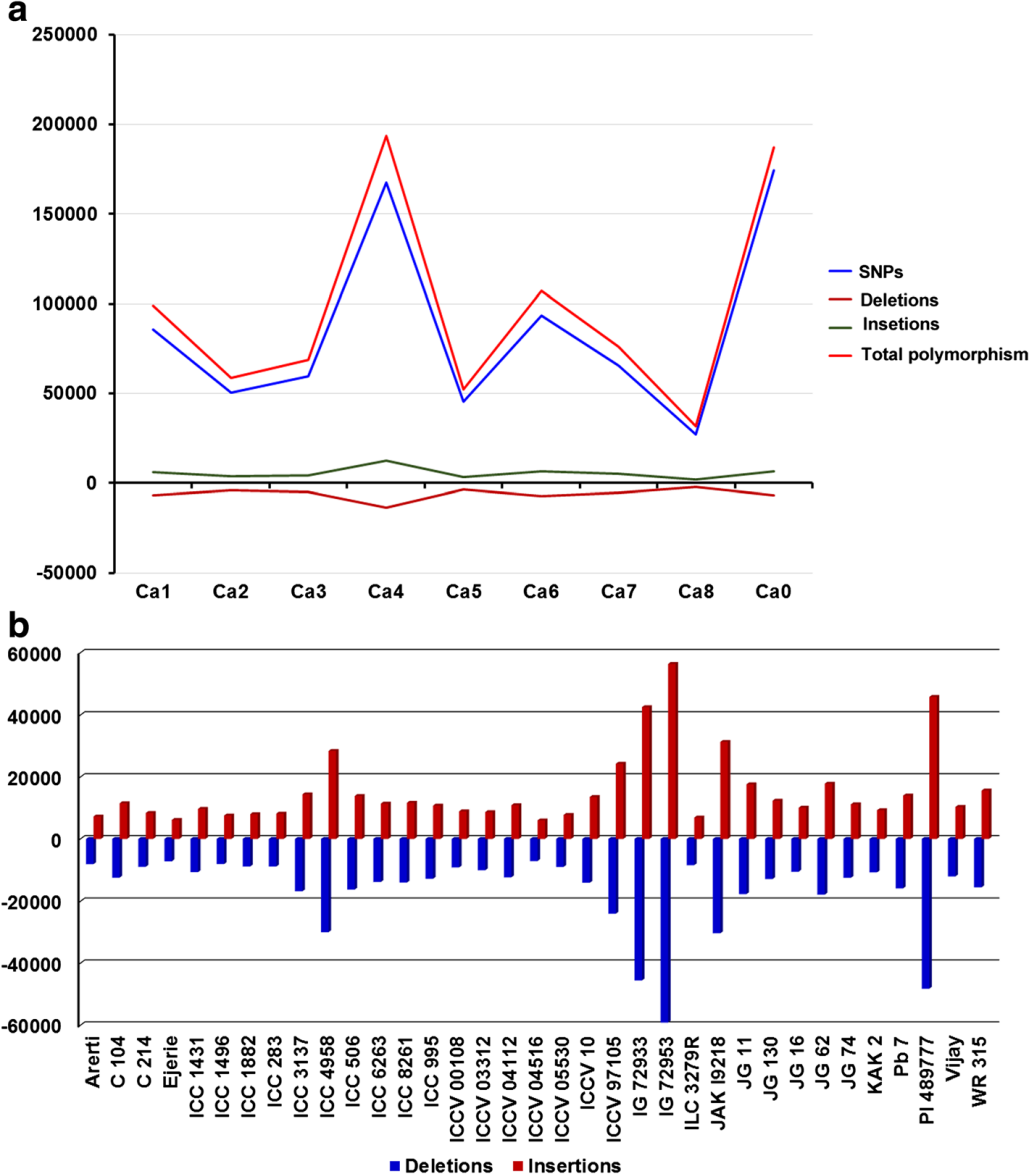
Genome-wide variations in 35 chickpea genotypes. **a** Distribution of SNPs, insertions, deletions and polymorphisms on eight pseudomolecules of chickpea. **b** Insertions and deletions identified in each chickpea genotype used in the present study

To determine the extent of sequence diversity among 35 chickpea genotypes, clean reads were aligned to the reference genome assembly of chickpea. As a result, a total of 2,058,566 SNPs were identified across all 35 genotypes re-sequenced (Additional file 3). Prior to this study, 51,632 SNPs were identified by 454 transcriptome sequencing of *Cicer arietinum* and *Cicer reticulatum* genotypes [18]. In addition, few hundreds of SNPs were also reported using Solexa ⁄ Illumina sequencing, amplicon sequencing of tentative orthologous genes (TOGs), mining of expressed sequence tags (ESTs) and sequencing of candidate genes [19–21]. WGRS approach has also been deployed in several crops for instance soybean [22], rice [23], pepper [24], maize [25] and tomato [26]. Among the SNPs on eight pseudomolecules (Ca1 to Ca8), most SNPs were identified on Ca4 (377,491) and the least on Ca8 (79,770), accounting for 18.34 and 3.88 % of the SNPs, respectively (Fig. 1a; Additional file 3). A total of 361,177 SNPs were identified on unanchored scaffolds and contigs (Ca0) accounting to 17.55 % of SNPs identified. The SNP density varied among pseudomolecules; Ca4 has the highest density (7.67 SNPs per Kb) and Ca0 had the lowest density (1.954 SNPs per Kb) (Additional file 3). Amongst the pseudomolecules, Ca4 was found to have maximum polymorphism rate (8.92/Kb), while Ca7 had least polymorphism rate (3.95/Kb). This study further re-affirms the results reported earlier [17] which may be due to presence of large repetitive regions in Ca4 pseudomolecule. The minimum density for exonic variants was observed on Ca7 (0.16 exon variants/Kb) while the maximum was found on Ca4 (0.36 exon variants/Kb) among the pseudomolecules (Additional file 3). Least density for exonic variants was 0.02 exon variants/Kb on Ca0. This means there were maximum changes in the coding regions of Ca4, in concurrence with the result of Varshney et al [17, 31]. The number of SNPs per genotype varied from 97,091 (ICCV 04516) to 1,001,744 (IG 72953) (Table 1). ICC 4958 among desi and ICC 8261 among kabuli genotypes were found to have maximum number of SNPs. The number of pair-wise SNPs were high between IG 72953 and IG 72933 (1,133,522 SNPs) and least between CDC-Frontier and ICCV 04516 (97,091) (Additional file 4). The number of SNPs reported in the study are higher compared to the previous studies [17, 27–30]. This may be due to diverse parental lines and wild genotypes used in the present study. The SNPs were categorized further into homozygous and heterozygous SNPs based on called SNPs in each genotype against the reference genome (Additional files 5 and 6). Maximum number of homozygous SNPs were identified in case of PI 489777 (606,413) and minimum in case of ICCV 04516 (57,432 SNPs). Among 35 genotypes maximum heterozygosity rate was observed in case of IG 72933 (0.84), while least heterozygosity rate was observed in case of PI 489777 (0.08). The mean heterozygosity rate was 0.36 across the 35 genotypes (Additional file 6).

**Table 1:** Summary of inter-and intra-genic SNPs in 35 chickpea genotypes

Genotype	Intergenic	No of intragenic SNPs								Others	
	No. of intergenic	Intron	Exon								
		No. of intronic SNPs	Non-synonymous					Synonymous			
			No. of non-synonymous coding	No. of non-synonymous start	No. of start lost	No. of stop gained	No. of stop lost	No. of synonymous coding	No. of synonymous stop		Total
Arerti	95,463	11,373	1,836	1	2	23	10	1,906	2	967	111,583
C 104	141,435	18,636	2,887	2	2	31	8	3,180	1	1,194	167,376
C 214	111,023	13,121	2,094	2	5	30	11	2,221	2	902	129,411
Ejerie	84,715	10,047	1,719	0	4	18	10	1,786	2	964	99,265
ICC 1431	126,604	16,306	2,456	1	5	25	12	2,828	3	1,007	149,247
ICC 1496	99,289	11,615	1,807	2	3	19	6	1,992	2	843	115,578
ICC 1882	107,060	12,517	1,936	3	3	23	11	2,101	5	882	124,541
ICC 283	109,146	13,296	1,943	1	3	24	10	2,130	2	861	127,416
ICC 3137	172,561	20,255	3,189	1	8	45	20	3,428	6	1,314	200,827
ICC 4958	292,686	35,022	5,159	3	13	58	19	5,884	8	1,951	340,803
ICC 506	169,681	19,028	2,977	4	7	32	15	3,292	6	1,358	196,400
ICC 6263	151,707	14,874	2,394	1	7	38	15	2,481	5	1,405	172,927
ICC 8261	151,627	16,987	2,586	1	7	30	10	2,802	4	1,290	175,344
ICC 995	146,193	15,647	2,428	1	8	25	14	2,748	3	1,247	168,314
ICCV 00108	110,493	11,327	1,442	2	3	19	6	1,459	2	927	125,680
ICCV 03312	118,283	12,341	1,996	0	5	30	10	2,142	3	1,123	135,933
ICCV 04112	133,378	17,305	2,383	1	7	30	8	2,712	4	1,093	156,921
ICCV 04516	84,366	8,806	1,385	2	3	20	7	1,565	0	937	97,091
ICCV 05530	107,685	12,448	1,942	1	7	31	13	2,055	3	995	125,180
ICCV 10	151,630	17,308	2,251	1	3	24	6	2,416	6	999	174,644
ICCV 97105	222,311	28,993	4,029	3	7	35	12	4,766	7	1,243	261,406
IG 72933	528,292	87,348	11,343	5	27	80	35	15,069	19	2,236	644,454
IG 72953	779,323	166,944	21,356	5	29	119	53	31,241	48	2,626	1,001,744
ILC 3279R	98,538	9,967	1,845	1	1	30	12	1,820	2	1,092	113,308
JAKI 9218	271,934	31,648	4,530	2	11	49	14	5,105	5	1,734	315,032
JG 11	180,028	21,190	2,559	2	5	27	7	2,761	4	1,202	207,785
JG 130	143,400	16,890	2,501	2	6	27	4	2,781	5	1,046	166,662
JG 16	126,104	15,899	2,238	2	5	27	11	2,437	5	887	147,615
JG 62	172,088	20,888	3,224	1	9	38	16	3,580	6	1,017	200,867
JG 74	146,466	18,427	2,743	1	5	27	12	3,147	6	1,154	171,988
KAK 2	125,250	13,638	2,041	1	5	30	9	2,253	3	1,130	144,360
PI 489777	530,227	99,288	12,742	5	24	94	32	17,737	24	2,032	662,205
Pb 7	169,234	18,338	2,800	2	6	37	11	3,010	7	1,245	194,690
Vijay	138,673	16,769	2,715	2	6	33	17	2,929	3	1,343	162,490
WR 315	158,812	20,911	3,102	2	4	32	17	3,550	6	934	187,370

### Insertions and deletions (*Indels*)

Insertions and deletions ranging from 1 bp to 58 bp were considered as *Indels* in the present study. In total, 292,588 *Indels* were identified across 35 chickpea genotypes (Additional file 3). The maximum number of deletions, 81,516 were 1 bp in length, while the least number of deletions (2) were of 52, 53, 56 and 57 bp in lengths. The maximum number of insertions were 78,678 with 1 bp length, while the minimum number was 1 with 58 bp length (Additional file 7). Of these *Indels*, 148,309 were the deletions and 144,279 were insertions. The density of deletions and insertions were 0.28 and 0.27 per Kb respectively across the genome (Additional file 3). Further, *Indel* analysis for each sample against the reference, CDC-Frontier, revealed the maximum *Indels* in IG 72953 (115,538), and minimum *Indels* in ICCV 04516 (13,146) respectively (Fig. 1b). When insertions to deletions ratio was calculated for each genotype, the maximum and minimum *indel* ratios were 1.03 and 0.81 in case of JAKI 9218 and ILC 3279 respectively. In JG 11, JG 62, WR 315 and ICCV 97105 the *Indel* ratio was ~1 (Additional file 8).

### Copy number variations (CNVs) and presence absence variations (PAVs)

CNVs and PAVs were determined in case of genes longer than 1 Kb. The gene ontology analysis was done using Swiss-prot and Trembl databases (http://www.ebi.ac.uk/uniprot). A non-redundant set of 9,732 genes were found duplicated across different genotypes and for 9,628 genes Uniprot IDs were retrieved and assigned. Out of these, 4,374 genes were found duplicated across just one of the samples making them line specific duplicated genes. Gene Ca_27299 was found duplicated across a maximum of 23 samples (Additional file 9). Ca_27299 with GO IDs GO:0016021; GO:0005886 was found to be Receptor-like protein 12 (AtRLP12) present to function for cell membrane. Duplicated genes ranged from 4 to 1135 amongst different genotypes. In JG 74, a maximum of 1,135 genes were found duplicated while the minimum number of genes (4) were duplicated in JG 62 (Additional file 9). Maximum number of defence related genes (27) were duplicated in a salt tolerant line ICC 1431.

Similarly, a non-redundant set of 205 genes were not found in any genotype. Uniprot IDs could be assigned for 198 genes. Amongst these, 134 genes were not present in any of the genotypes suggesting line specific gene deletion (Additional file 10). The gene Ca_17015 was absent in eight genotypes, however, it is an uncharacterized protein. Ca_13947 was not present in 7 genotypes and its putative function was Pentatricopeptide repeat-containing protein belonging to PPR family, PCMP-E subfamily. The PAVs result depicted that there were no genes deleted in ICCV 03312, IG 72953 and PI 489777 (Additional file 10). A maximum of 32 genes were found deleted in JG 62 followed by 30 genes in ICCV 00108.

###  Miscellaneous variations

In addition to above variations, an effort was made to identify miscellaneous variations like inter-chromosomal variations (CTX), intra-chromosomal variations (ITX) and inversions (INV). Of 539 miscellaneous variations, 339, 138 and 62 were CTX, ITX and INV respectively. To further avoid false positives, we have used stringent cutoff of 99. As a result 110 miscellaneous variations were identified on eight pseudomolecules (Table 2). CTX were in the range of 273 bp to 667 bp spread over Ca3 (22), Ca6 (16), Ca4 (10), Ca1 (7), Ca5 (7) and Ca7 (3). ITX were in the range of 86 bp to 3.81 Mbp spread over Ca2 (11), Ca4 (4), Ca5 (4), Ca7 (4), Ca8 (3) and Ca3 (2). While INV were in the range of 30 bp to 4.76 Mbp predominantly on Ca6 (8), followed by Ca4 (2), Ca7 (2) and Ca2 (1).

**Table 2:** Distribution of miscellaneous variations on eight pseudomolecules of chickpea

Type of miscellaneous variation*	Pseudomolecules								Total
	Ca1	Ca2	Ca3	Ca4	Ca5	Ca6	Ca7	Ca8	
CTX	4	2	2	4	9	10	30	6	67
INV	0	1	0	2	0	8	2	0	13
ITX	0	11	2	4	4	2	4	3	30
Total	4	14	4	10	13	20	36	9	110

*CTX – inter-chromosomal translocations, ITX – intra-chromosomal, INV – inversions and translocation

### Line specific variations

**Fig. 2:**
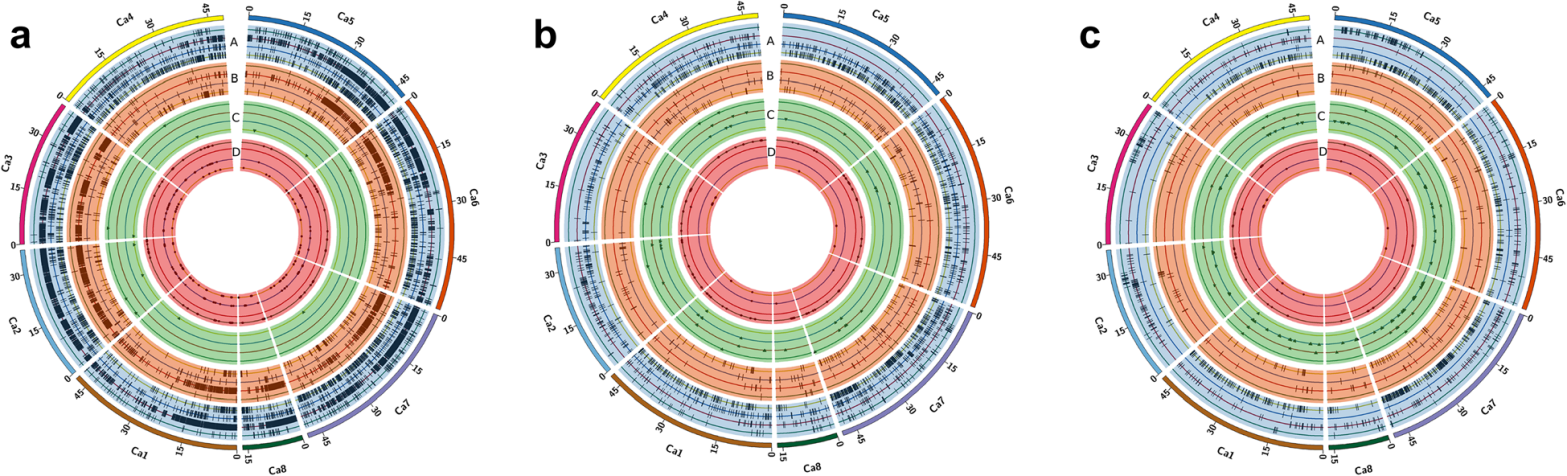
Genome-wide variations identified in chickpea genotypes resistant or susceptible to important biotic stresses. Circos diagrams represent line specific SNPs, *Indels*, gene deletions and duplications. Each circos represents eight chickpea pseudomolecules and consists of four concentric rings where A represents SNPs, B represents Indels, C represents deletion and D represents duplication events. The deletion events are marked with triangles in green ring and circles represent duplications in red. **a** Distribution of variations among *Helicoverpa* resistant and susceptible genotypes. The green, red, blue, and yellow color inside all rings represents Vijay, IG 72953, ICC 506 and ICC 3137 respectively. **b** Distribution of variations among *Fusarium* wilt resistant and susceptible genotypes. The green, red, blue, and yellow color inside all rings represents C 104, JG 62, WR 315 and ICCV 05530 respectively. **c** Distribution of variations among *Ascochyta* blight resistant and susceptible genotypes. The green, red, blue, and yellow color inside all rings represents ICCV 04516, JG 62, Pb 7 and ICCV 05530 respectively

A total of 164,856 unique line specific variations including 144,888 SNPs and 19,968 *Indels* were observed among 35 chickpea genotypes studied. Maximum number of line specific variations, 78,320, were observed in PI 489777 (68,799 SNPs and 9,521 *Indels*), and followed by 62,808 in IG 72953 (55,393 SNPs and 7,415 *Indels*) (Fig. 2a; Additional file 11). We further compared line specific variations among parental genotypes that segregate for abiotic stresses (like drought, salinity) and biotic stresses (like *Fusarium* wilt, *Ascochyta* blight, *Botrytis* grey mould). Although larger number of line specific SNPs and *Indels* were identified in the case of *Helicoverpa* resistant wild species genotype IG 72953, interestingly no species specific deletion and duplication of genes were identified (Fig. 2a). On contrary, in the case of parental genotypes of mapping population segregating for *Helicoverpa* resistance (ICC 506 × ICC 3137) the number of line specific SNPs and *Indels* differed significantly. Similarly, large number of line specific variations among parental lines of mapping populations segregating for *Ascochyta* blight (Fig. 2b), *Fusarium* wilt (Fig. 2c), *Botrytis* grey mould and salinity (Additional files 12 and 13), were identified that can be used for developing high density genetic maps, trait mapping as well as for marker-assisted selection. Among 35 chickpea genotypes, the line specific variations were < 100 in case of ICC 1496, ICCV 00108, Pb 7, JG 130, ICC 4958, JAKI 9218 and C 214. Among 35 genotypes, interestingly no line specific variation was observed in the case of C 214. Further, the number of line specific variations were in the range of ~100 to ~5,000 in case of remaining 26 genotypes (Additional file 13). The maximum percentage of line specific variations found in coding regions in ICC 1496 (21 %) followed by ICCV 97105 (12 %) (Additional file 14). The mean of the line specific variations in the coding region was found 6.4 %, while none of the line specific variations in coding regions were observed in the case of ICC 4958 and JAKI 9218. When the frame shift, start lost, stop gained and stop lost mutations were summed up and their percentage were calculated out of the total variations in coding regions, the maximum of 33.33 % was observed in Pb 7 genotype, while there were none in 8 genotypes (ICCV 04112, ICCV 04516, C 104, ICCV 00108, JG 11, JG 130, ICCV 10 and ICC 1431) (Additional file 15). Among the parental lines of MAGIC population, line specific SNPs, line specific deletions and line specific insertions were high in case of JG 11. Further, gene deletions ranged from 8 (JAKI 9218) to 30 (ICCV 00108), while gene duplications ranged from 17 (JG 130) to 1,120 (JAKI 9218) (Table 3). Overall large variation is evident at genome level in case of parental lines of MAGIC population. The main purpose of developing MAGIC populations is to create and harness the genetic diversity for crop improvement. In summary, the MAGIC lines developed from these lines will possess tremendous variation that can be used for allele mining and gene discovery. The line specific variants were further annotated using Uniprot repository. The annotation revealed the effect of these line specific variants on a number of transcription factors and their regulators like zinc finger protein, bHLH, WRKY, F-Box, bZIP, PHD, SCREAM and MADS box, etc. Along with TFs, disease resistace NB-LRR protein, heat shock proteins, DNA- damage repair proteins, nodulation signaling pathway related proteins were also affected (Additional file 16).

**Table 3:** Line specific variations identified among parental lines of the chickpea MAGIC population

Genotype	Total SNPs	Line specific SNPs	Total deletions	Line specific deletions	Total insertions	Line specific insertions	Genes deleted	Genes duplicated
ICC 4958	340,803	3	29,976	1	28,429	0	23	47
ICCV 00108	125,680	49	9,217	6	8,907	2	30	323
ICCV 10	174,644	219	14,137	20	13,534	22	14	21
ICCV 97105	261,406	120	24,016	6	24,274	13	18	42
JAKI 9218	315,032	1	30,320	0	31,339	0	8	1,120
JG 11	207,785	496	17,690	36	17,640	31	21	25
JG 130	166,662	13	12,936	2	12,342	3	25	17
JG 16	147,615	95	10,579	10	10,094	6	27	73

### Annotation of genome-wide variations

In general, premature stop, frame-shift and presence/absence variations lead to genetic load by disabling the gene functions which may lead to inbreeding depression. Hence, we annotated the genome-wide variations. Among 2,351,154 variations, 38,078 were mis-sense, 352 were non-sense and 50,290 were the silent mutations (Additional file 17). However, large variations (1,924,406) were in intergenic region. Of 419,262 variations detected in the genic regions, 328,171 were intronic variations and 91,091 were exonic variations (Additional file 18). Out of these exonic variations, 37,875 were non-synonymous substitutions. On comparing the distribution of SNPs across genomic regions, SNPs were most abundant in intergenic regions (81.85 %) and the proportion of SNPs were high in the introns (13.96 %) than exons (3.87 %). Genetic variant annotation and effect prediction tool was used to predict the effect of all SNPs, homozygous SNPs and heterozygous SNPs identified among all 35 chickpea genotypes (Additional files 18, 19, and 20). The number of SNPs leading to stop-gain or non-sense mutations among different genotypes ranged from 18 (Ejerie) to 119 (IG 72953). The *Indels* were studied for their affects on the genome for each of the genotypes. Maximum insertions affecting the genic regions was seen in IG 72953 (21.54 %) while the least in ICCV 00108 (12.23 %) (Additional file 21). Similarly, maximum deletions occurring in the genic regions were observed in IG 72953 (21.16 %) and the minimum of 12.90 % in JAKI 9218 (Additional file 22).

We identified 373 variations in the “*QTL-hotspot*” region on Ca4, reported earlier to enhance the drought tolerance in chickpea [6, 31] (Additional file 20). Among these variations, notably two codon insertions were found in Ca_04570 (present in “*QTL-hotspot-b*”) belonging to 7S seed storage gene family and reported to enhance seed size [32]. In addition we also identified 38 variations that were non-synonymous coding affecting a total of 17 genes with functions like heat stress transcription factor A-6b, EPF-like protein 4 and Early light-induced protein (chloroplastic ELIP) (Additional file 23).

## Conclusion

The genome-wide variations identified in the present study can be used for developing high density SNP arrays for genetics and breeding applications. Further, large number of line specific variations among the wild accessions indicate that the wild chickpea has much more diverse genepool than the cultivated chickpea, thus may contain useful genetic resources for chickpea improvement.

## Methods

### Plant material

Thirty five chickpea genotypes, used in the study, their pedigree, origin, market class and salient features are presented in Additional file 1.

### DNA isolation

Genomic DNA was isolated from all 35 chickpea genotypes from 10-days old etiolated seedlings as described earlier [33]. The quality of DNA was checked on 0.8 % agarose gel. The Qubit® 2.0 Fluorometer (Life technologies, Thermo Fisher Scientific Inc. USA) was used for quantification of DNA.

### Library construction and sequencing

Approximately, 1 μg DNA from each sample was used for construction of a MiSeq sequencing library using TruSeq DNA sample Prep kit LT, (set A) FC-121-2001 (Illumina, San Diego, CA, USA) according to manufacturer’s protocol. Briefly, column purified genomic DNA was sheared using Bioruptor® NGS (Diagenode, Belgium). The size of fragmented DNA was determined on 1.2 % agarose gel and was found within the range of 200–1000 bp. After shearing, the end-repair was performed to convert the overhangs into blunt ends followed by adenylate 3’ ends. Subsequently, indexed adaptors were ligated to the ends of the DNA fragments to make them ready for hybridization onto the flow cell. Size selection was performed using l E-Gel® Size™ 2 % agarose precast gels (Invitrogen) to get a target insert size of about 400 bp and purified. PCRs (9 cycles) were performed to enriched the size selected DNA fragments having Illumina adapters on both the ends. The size distribution of amplified DNA library was checked on an Agilent Technologies 2100 bioanalyzer using Agilent high sensitivity DNA chip.

Denatured and diluted libraries were sequenced on Illumina MiSeq benchtop sequencer (Illumina, San Diego, CA, USA) using MiSeq Reagent Kit v2 (300-cycles) to generate 150 bases paired-end reads. Data was demultiplexed on the MiSeq instrument automatically and sample wise zipped FASTQ files were generated.

### Data filtering and alignment

The raw data generated for each line were cleaned and trimmed using sickle version 1.200 (https://github.com/najoshi/sickle). The cleaned data were aligned on to the reference chickpea genome [17] using Bowtie 2 [34]. The alignment data were further filtered to retain the reads mapped to only one region along the genome. The reads with a minimum mapping quality of 30, were used for further analysis. Base Quality Score Recalibration (BQSR) and InDel Realignment components of The Genome Analysis Toolkit (GATK, v 3.1-1) [35], multiple utilities from Picard (v1.102) (http://broadinstitute.github.io/picard/) were used for post-processing of the bam files.

### Identification of genome-wide variations

The alignment files generated after the above mentioned stringent criteria were used for variant discovery with GATK program. A position was reported as a variant for a genotype if the phred quality score > 30 supported by a minimum read depth of 5. Variants with less than 5 bp flanking distance were filtered out. Distribution of DNA polymorphisms was assessed by calculating their frequency in a window size of 100 Kb along each pseudomolecule. For identification of effects of synonymous and non-synonymous SNPs and *Indels*, SnpEff program [36] was used. In-house Perl scripts were used to analyze the distribution of the variations (SNPs and Indels) across the genome. Line specific variations were reported only if the variation was present in only one genotype and the reference allele was present in rest of the genotypes. The line specific variations were further studied for their effects on the coding sequences and these variations were assigned GO IDs with the information retrieved from UniProtKB for the genes showing hits to UniProt IDs. Circos diagrams were used to plot line specific variations [37].

CNVnator was used to find the CNVs with an e-value cutoff of 1e-05 and results were annotated with genes ≥ 1,000 bp in length [38]. False positives were eliminated by excluding the CNVs discovered by mapping the reads to CDC Frontier. PAVs were determined based on the sequencing depth (<10 % was considered as absence variation and >50 % was considered as presence variation). For determining miscellaneous variations like ITX, CTX and INV, paired-end reads from each sample were aligned to the reference genome (CDC Frontier) using Bowtie 2 with discordant flags and in end-to-end mapping mode. Picard (v1.102) (http://broadinstitute.github.io/picard/) was used to set the read group information on the alignment files and sorted by coordinate position using SAMtools (v0.1.19+) [39]. Breakdancer (v1.1.2) [40] was used to detect miscellaneous variations. The miscellaneous variations showing the presence of reads from control were omitted as they are considered as false positives. The miscellaneous variations were filtered by score equal to 99 thereby selecting a highly confident set of miscellaneous variations. Statistics of the miscellaneous variations were summarized by type in R (v3.1). Breakdancer provides only the breakpoint coordinates, within which the detected miscellaneous variations reside. So, in order to find the exact nucleotide coordinates of the miscellaneous variations, the reads supporting each of the miscellaneous variations were looked-up in the alignment files. This was performed using an inhouse tool in C++.

### Availability of supporting data

The data sets supporting the results of this article are available at http://ceg.icrisat.org/publicdomain.html.

## Additional files

Additional file 1:
**Details on geographic origin, market class and pedigree information of 35 chickpea genotypes used in the study.** (DOCX 17 kb) Additional file 2:
**Summary of data generated for 35 chickpea genotypes and aligned to reference genome of chickpea (CDC Frontier).** (DOC 70 kb) Additional file 3:
**Distribution of SNPs,** ***Indels*** **and their effects in the chickpea genome (Ca1-Ca8). ** (DOCX 14 kb) Additional file 4:
**Pairwise SNPs identified among 35 chickpea genotypes used in this study.** (XLSX 17 kb) Additional file 5:
**Homozygous and heterozygous SNPs identified in each chickpea genotype used in the study.** Maximum heterozugous SNPs are evident in IG 72933 while homozygous SNPs in PI 489777. (TIF 309 kb) Additional file 6:
**Summary of homozygous and heterozygous SNPs identified in 35 chickpea genotypes.** (XLSX 11 kb) Additional file 7:
**Summary of** ***Indel*** **lengths and their respective counts across the genome.** (XLSX 9 kb) Additional file 8:
**Summary of** ***Indel*** **ratio among 35 chickpea genotypes re-sequenced.** (XLSX 12 kb) Additional file 9:
**Gene duplications observed in 35 chickpea genotypes.** (XLSX 2979 kb) Additional file 10:
**Gene deletion detected in 35 chickpea genotypes used in this study.** (XLSX 67 kb) Additional file 11:
**Genome-wide variations identified in chickpea genotypes resistant or susceptible to** ***Botrytis*** **grey mould.** Circos diagram represents line specific variations. Each circos represents eight chickpea pseudomolecules and consists of four concentric rings where A represents SNPs, B represents *Indels*, C represents deletion and D represents duplication events. The deletion events are marked with triangles in green ring and circles represent duplications in red. The green, red, blue, and yellow color inside all rings represents ICC 1496 (resistant), JG 62 (susceptible), ICCV 10 (susceptible), ICCV 05530 (resistant) respectively. (PNG 3357 kb) Additional file 12:
**Genome-wide variations identified in chickpea genotypes tolerant or susceptible to salinity.** Circos diagram represents line specific variations. Each circos represents eight chickpea pseudomolecules and consists of four concentric rings where A represents SNPs, B represents *Indels*, C represents deletion and D represents duplication events. The deletion events are marked with triangles in green ring and circles represent duplications in red. The green, red, blue, and yellow color inside all rings represents ICC 1431 (tolerant), JG 62 (tolerant), ICC 6263 (susceptible), JG 11 (tolerant) respectively. (PNG 3400 kb) Additional file 13:
**Summary of line specific variations.** (XLSX 11 kb) Additional file 14:
**Summary of line specific variations in coding region identified in each chickpea genotype.** (XLSX 12 kb) Additional file 15:
**Summary of line specific variations leading to stop sites.** (XLSX 11 kb) Additional file 16:
**Gene ontology and their effects for the line specific variations in 35 genotypes.** (XLSX 727 kb) Additional file 17:
**Summary of all variant effects predicted by SNPEff.** (XLSX 12 kb) Additional file 18:
**Classification of all SNPs using SNPEff program.** (XLSX 10 kb) Additional file 19:
**Classification of heterozygous SNPs using SNPEff program in 35 chickpea genotypes.** (XLSX 12 kb) Additional file 20:
**Classification of homozygous SNPs using SNPEff program in 35 chickpea genotypes.** (XLSX 12 kb) Additional file 21:
**Summary of effects for the insertions.** (DOCX 15 kb) Additional file 22:
**Summay of effects for the deletions.** (DOCX 15 kb) Additional file 23:
**Summary of variations identified in the “** ***QTL-hotspot*** **” region reported by Varshney et al.** [6, 8]. (XLSX 91 kb)

## Abbreviations

WGRS: Whole genome re-sequencing
DEL: Deletions
INS: Insertions
CTX: Inter-chromosomal translocations
ITX: Intra-chromosomal translocations
CNV: Copy number variations
