# Supplementary material for: Whole genome re-sequencing reveals genome-wide variations among parental lines of 16 mapping populations in chickpea (Cicer arietinum L.)
Source: BMC Plant Biol. 2016 Jan 27;16(Suppl 1):10. doi: 10.1186/s12870-015-0690-3 (PMC4895712; doi:10.1186/s12870-015-0690-3)
Supplement: Additional file 1: — Details on geographic origin, market class and pedigree information of 35 chickpea genotypes used in the study. (DOCX 17 kb) [file 12870_2015_690_MOESM1_ESM.docx]

**Additional File 1: Details on 35 select chickpea inbred genotypes used in the study**

| **Genotype** | **Pedigree/parentage** | **Market class** | **Origin** | **Species** | **features** |
| --- | --- | --- | --- | --- | --- |
| Arerti | X87TH186/ICC 14198 × FLIP 82-150C | Kabuli | ICRISAT | *C. arietinum* | Drought susceptible |
| C 104 | - | Desi | India | *C. arietinum* |  |
| C 214 | - | Desi | India | *C. arietinum* | Susceptible to *Fusarium* wilt |
| Ejerie | X94TH71/FLIP 87-59 C × UC 15 | Kabuli | ICRISAT | *C. arietinum* | Drought susceptible |
| ICC 1431 | P 1260-1 | Desi | India | *C. arietinum* | Salt tolerant |
| ICC 1496 | P 1292-1 | Desi | India | *C. arietinum* | *Botrytis* grey mould resistant |
| ICC 1882 | P 1506-4 | Desi | India | *C. arietinum* | Drought susceptible |
| ICC 283 | P 223-1 | Desi | India | *C. arietinum* | Drought susceptible |
| ICC 3137 | P 3659-2 | Desi | Iran | *C. arietinum* | Susceptible to *Helicoverpa armigera* |
| ICC 4958 | JGC1 | Desi | India | *C. arietinum* | Drought tolerant genotype found promising in Ethiopia, Kenya and India; |
| ICC 506 | P 386 | Desi | India | *C. arietinum* | Resistant to *Helicoverpa armigera* |
| ICC 6263 | NEC 142 | Kabuli | Russia & CISs | *C. arietinum* | Salt sensitive |
| ICC 8261 | NEC 2430 | Kabuli | Turkey | *C. arietinum* | Donor parent for drought related root traits |
| ICC 995 | P 809 | Desi | Mexico | *C. arietinum* | Protein content |
| ICCV 00108 | IG 9216 x ICCV 10 | Desi |  | *C. arietinum* | Farmer-preferred elite line identified and released in Tanzania and Kenya |
| ICCV 03312 | - |  | ICRISAT | *C. arietinum* | - |
| ICCV 04112 | ICCX-970047-BP-BP-P72-BP-BP | Desi | ICRISAT | *C. arietinum* | MARS population development |
| ICCV 04516 | ICC 1069 x NEC 138-2 | Desi | ICRISAT | *C. arietinum* | AB resistant line tolerant to Hissar race |
| ICCV 05530 | Pant G-114 x ICC 3935 | Desi | ICRISAT | *C. arietinum* | Mapping pop. ABR, BGM, FW |
| ICCV 10 | P 1231 × P 1265 | Desi | India | *C. arietinum* | Widely adapted drought tolerant cultivar found promising in India and Kenya |
| ICCV 97105 | ICCV 10 × GL 769 | Desi | ICRISAT | *C. arietinum* | Farmer-preferred elite line identified and released in Tanzania and ready for release in Kenya |
| IG 72933 | wild | Desi | Unknown | *C. reticulatum* | *Helicoverpa* resistant |
| IG 72953 | wild | Desi | Turkey | *C. reticulatum* | *Helicoverpa* resistant |
| ILC 3279R | Breeding line/Cultivar | Kabuli |  | *C. arietinum* | Donor parent for *Aschochyta* blight resistance |
| JAKI 9218 | (ICCC 37 × GW 517) × ICCV 17 | Desi | India | *C. arietinum* | Farmer-preferred cultivar in central and southern India |
| JG 11 | (Phule G-5 × Narsingpur Bold) × ICCC 37 | Desi | India | *C. arietinum* | Farmer-preferred cultivar in southern India and also performing well in Kenya |
| JG 130 | ICCC 42 × BG 256 | Desi | India | *C. arietinum* | Farmer-preferred cultivars from central India |
| JG 16 | ICCC 42 × ICCV 88506) × (KPG 59 × JK74) | Desi | India | *C. arietinum* | Farmer-preferred cultivar in northern and central India |
| JG 62 |  | Desi | India | *C. arietinum* |  |
| JG 74 |  |  |  |  |  |
| KAK 2 | ICCV 2 × Surutato 77) × ICC 7344 | Kabuli | India | *C. arietinum* | Early maturity with high yield and resistant to *Fusarium* wilt |
| Pb 7 | - | Desi | India | *C. arietinum* |  |
| PI 489777 | - | wild | Turkey | *C. reticulatum* | Resistant to *Helicoverpa armigera* |
| Vijay | - | Desi |  | *C. arietinum* |  |
| WR 315 | - | Desi |  | *C. arietinum* | Resistant to *Fusarium* wilt |
