# Supplementary material for: Whole genome re-sequencing reveals genome-wide variations among parental lines of 16 mapping populations in chickpea (Cicer arietinum L.)
Source: BMC Plant Biol. 2016 Jan 27;16(Suppl 1):10. doi: 10.1186/s12870-015-0690-3 (PMC4895712; doi:10.1186/s12870-015-0690-3)
Supplement: Additional file 2: — Summary of data generated for 35 chickpea genotypes and aligned to reference genome of chickpea (CDC Frontier). (DOC 70 kb) [file 12870_2015_690_MOESM2_ESM.doc]

**Additional File** 2: Summary of data generated on 35 select chickpea inbred lines and aligned to reference genome CDC Frontier

| **Genotype** | **No of reads generated** | **Total Bases generated (bp)** | **No of high quality reads** | **No of reads aligned** | **No of reads uniquely aligned** | **Alignment (%)** | **Unique Alignment (%)** | **Genome coverage** | **Mean depth** |
| --- | --- | --- | --- | --- | --- | --- | --- | --- | --- |
| Arerti | 18,759,680 | 4,679,150,537 | 18,447,188 | 16,919,504 | 10,301,796 | 91.72 | 55.84 | 82.17 | 7.62 |
| C 104 | 19,567,320 | 4,881,104,888 | 19,229,040 | 17,753,049 | 10,747,399 | 92.32 | 55.89 | 82.19 | 7.98 |
| C 214 | 16,524,566 | 4,123,174,185 | 16,245,882 | 14,943,062 | 9,025,637 | 91.98 | 55.56 | 81.24 | 6.72 |
| Ejerie | 17,924,884 | 4,470,839,445 | 17,632,667 | 16,311,989 | 9,697,851 | 92.51 | 55.00 | 81.91 | 7.36 |
| ICC 1431 | 17,718,020 | 4,420,081,242 | 17,438,729 | 15,992,922 | 9,603,736 | 91.71 | 55.07 | 81.71 | 7.25 |
| ICC 1496 | 14,114,248 | 3,521,780,369 | 13,896,645 | 12,765,493 | 7,778,952 | 91.86 | 55.98 | 80.64 | 5.79 |
| ICC 1882 | 15,260,764 | 3,808,078,782 | 14,966,231 | 13,862,632 | 8,243,224 | 92.63 | 55.08 | 81.03 | 6.21 |
| ICC 283 | 15,262,898 | 3,808,703,401 | 15,025,367 | 13,845,414 | 8,291,801 | 92.15 | 55.19 | 80.94 | 6.23 |
| ICC 3137 | 19,423,590 | 5,804,129,544 | 18,746,951 | 17,208,606 | 10,720,920 | 91.79 | 57.19 | 82.85 | 8.73 |
| ICC 4958 | 45,053,978 | 9,105,790,900 | 43,565,446 | 39,873,460 | 23,716,646 | 91.53 | 54.44 | 84.04 | 14.26 |
| ICC 506 | 18,625,982 | 5,564,153,846 | 18,117,051 | 16,636,951 | 10,275,090 | 91.83 | 56.72 | 82.76 | 8.50 |
| ICC 6263 | 17,272,430 | 5,159,563,816 | 16,863,332 | 15,506,932 | 9,690,227 | 91.96 | 57.46 | 82.61 | 7.93 |
| ICC 8261 | 16,594,522 | 4,960,904,577 | 16,136,716 | 14,903,804 | 9,360,729 | 92.36 | 58.01 | 82.61 | 7.58 |
| ICC 995 | 16,134,000 | 4,822,932,526 | 15,686,308 | 14,422,412 | 8,905,533 | 91.94 | 56.77 | 82.20 | 7.33 |
| ICCV 00108 | 25,258,824 | 3,791,864,934 | 24,799,971 | 22,977,290 | 13,671,095 | 92.65 | 55.13 | 81.58 | 6.45 |
| ICCV 03312 | 14,751,308 | 4,405,583,166 | 14,392,310 | 13,180,927 | 8,133,481 | 91.58 | 56.51 | 81.55 | 6.81 |
| ICCV 04112 | 15,582,618 | 4,654,315,379 | 15,185,845 | 13,820,632 | 8,629,512 | 91.01 | 56.83 | 82.01 | 7.12 |
| ICCV 04516 | 16,727,518 | 4,993,034,828 | 16,005,579 | 14,747,040 | 8,843,461 | 92.14 | 55.25 | 81.75 | 7.04 |
| ICCV 05530 | 15,287,850 | 4,552,165,876 | 14,960,768 | 13,561,495 | 8,365,799 | 90.65 | 55.92 | 81.27 | 7.03 |
| ICCV 10 | 34,613,120 | 5,188,524,710 | 33,902,317 | 31,696,707 | 18,957,142 | 93.49 | 55.92 | 82.97 | 8.84 |
| ICCV 97105 | 89,468,100 | 10,681,959,520 | 77,716,712 | 73,137,409 | 33,980,973 | 94.11 | 43.72 | 84.00 | 16.40 |
| IG 72933 | 18,778,498 | 5,590,331,947 | 18,242,852 | 16,655,770 | 10,179,246 | 91.30 | 55.80 | 82.68 | 8.57 |
| IG 72953 | 17,056,610 | 5,081,707,309 | 16,456,148 | 14,841,035 | 8,955,202 | 90.19 | 54.42 | 80.25 | 7.56 |
| ILC 3279R | 17,825,460 | 5,301,667,320 | 17,410,543 | 15,883,520 | 9,747,051 | 91.23 | 55.98 | 82.41 | 8.23 |
| JAKI 9218 | 106,335,742 | 12,756,473,009 | 92,465,227 | 87,487,094 | 40,072,407 | 94.62 | 43.34 | 84.18 | 20.04 |
| JG 130 | 35,138,818 | 5,272,434,481 | 34,587,771 | 32,023,627 | 18,316,549 | 92.59 | 52.96 | 83.01 | 8.98 |
| JG 16 | 28,738,482 | 4,312,870,046 | 28,305,474 | 26,445,080 | 15,649,655 | 93.43 | 55.29 | 82.15 | 7.42 |
| JG 62 | 73,496,400 | 7,423,136,400 | 67,397,379 | 64,228,222 | 24,819,306 | 95.30 | 36.83 | 83.23 | 12.18 |
| JG 74 | 18,754,914 | 5,586,709,863 | 18,035,141 | 16,474,011 | 9,939,330 | 91.34 | 55.11 | 81.78 | 7.88 |
| KAK 2 | 15,504,622 | 4,631,161,234 | 14,995,047 | 13,743,511 | 8,638,069 | 91.65 | 57.61 | 81.91 | 7.06 |
| Pb 7 | 20,093,340 | 5,985,093,811 | 19,359,575 | 17,806,239 | 11,027,941 | 91.98 | 56.96 | 82.66 | 8.57 |
| PI 489777 | 17,860,416 | 5,322,466,470 | 17,293,121 | 15,664,692 | 9,248,052 | 90.58 | 53.48 | 79.76 | 8.00 |
| Vijay | 17,759,280 | 5,293,509,004 | 17,087,535 | 15,728,306 | 9,717,554 | 92.05 | 56.87 | 82.18 | 8.05 |
| WR 315 | 74,363,384 | 7,510,701,784 | 59,593,887 | 56,353,383 | 21,767,933 | 94.56 | 36.53 | 83.14 | 10.68 |
| JG 11 | 31,499,366 | 4,726,175,934 | 31,035,068 | 28,443,060 | 16,544,593 | 91.65 | 52.52 | 82.63 | 7.98 |
| Total | 973,131,552 | 192,192,275,083 | 911,225,823 | 845,845,280 | 461,563,892 |  |  |  |  |
| Average | 27,803,759 | 5,491,207,860 | 26,035,024 | 24,167,008 | 13,187,540 | 92.18 | 53.92 | 82.17 | 8.58 |
