# Supplementary material for: Whole genome re-sequencing reveals genome-wide variations among parental lines of 16 mapping populations in chickpea (Cicer arietinum L.)
Source: BMC Plant Biol. 2016 Jan 27;16(Suppl 1):10. doi: 10.1186/s12870-015-0690-3 (PMC4895712; doi:10.1186/s12870-015-0690-3)
Supplement: Additional file 3: — Distribution of SNPs, Indels and their effects in the chickpea genome (Ca1-Ca8). (DOCX 14 kb) [file 12870_2015_690_MOESM3_ESM.docx]

| **Additional File 3: Distribution of SNPs, indels and their effects on chickpea genome** | | | | | | | | |  |  |  | |  |  |  |  |
| --- | --- | --- | --- | --- | --- | --- | --- | --- | --- | --- | --- | --- | --- | --- | --- | --- |
| **Pseudomolecule** | **SNPs** | **SNPs/Kb** | **Insertions** | **Insertions/Kb** | **Deletions** | **Deletions/Kb** | **Indels** | **Indels/Kb** | **Polymorphisms** | **Polymorphisms/ Kb** | **Genic** | | | **Intergenic** | **Others** | **Total** |
|  |  |  |  |  |  |  |  |  |  |  | **Exon** | **Intron** | |  |  |  |
| **Ca1** | 249,850 | 5.17 | 18,308 | 0.38 | 19,230 | 0.40 | 37,538 | 0.78 | 287,388 | 5.94 | 11,787 | 42,767 | | 232,833 | 1 | 287,388 |
| **Ca2** | 138,528 | 3.78 | 9,899 | 0.27 | 10,009 | 0.27 | 19,908 | 0.54 | 158,436 | 4.32 | 7,090 | 27,007 | | 124,322 | 17 | 158,436 |
| **Ca3** | 202,170 | 5.06 | 14,960 | 0.37 | 15,639 | 0.39 | 30,599 | 0.77 | 232,769 | 5.82 | 10,716 | 37,885 | | 184,157 | 11 | 232,769 |
| **Ca4** | 377,491 | 7.67 | 30,405 | 0.62 | 31,002 | 0.63 | 61,407 | 1.25 | 438,898 | 8.92 | 18,126 | 65,425 | | 355,342 | 5 | 438,898 |
| **Ca5** | 184,671 | 3.83 | 13,585 | 0.28 | 14,131 | 0.29 | 27,716 | 0.58 | 212,387 | 4.41 | 10,358 | 35,645 | | 166,364 | 20 | 212,387 |
| **Ca6** | 294,917 | 4.96 | 21,162 | 0.36 | 22,079 | 0.37 | 43,241 | 0.73 | 338,158 | 5.69 | 14,461 | 51,732 | | 271,948 | 17 | 338,158 |
| **Ca7** | 169,992 | 3.47 | 11,820 | 0.24 | 11,704 | 0.24 | 23,524 | 0.48 | 193,516 | 3.95 | 8,323 | 29,227 | | 155,959 | 7 | 193,516 |
| **Ca8** | 79,770 | 4.84 | 6,526 | 0.40 | 6,856 | 0.42 | 13,382 | 0.81 | 93,152 | 5.65 | 5,569 | 20,248 | | 67,335 | 0 | 93,152 |
| **Ca0** | 361,177 | 1.95 | 17,614 | 0.10 | 17,659 | 0.10 | 35,273 | 0.19 | 396,450 | 2.14 | 4,661 | 18,235 | | 366,146 | 7,408 | 396,450 |
| **Total** | 2,058,566 | 3.87 | 144,279 | 0.27 | 148,309 | 0.28 | 292,588 | 0.55 | 2,351,154 | 4.42 | 91,091 | 328,171 | | 1,924,406 | 7,486 | 2,351,154 |
