# Supplementary figures and images for: Whole genome re-sequencing reveals genome-wide variations among parental lines of 16 mapping populations in chickpea (Cicer arietinum L.)
Source: BMC Plant Biol. 2016 Jan 27;16(Suppl 1):10. doi: 10.1186/s12870-015-0690-3 (PMC4895712; doi:10.1186/s12870-015-0690-3)

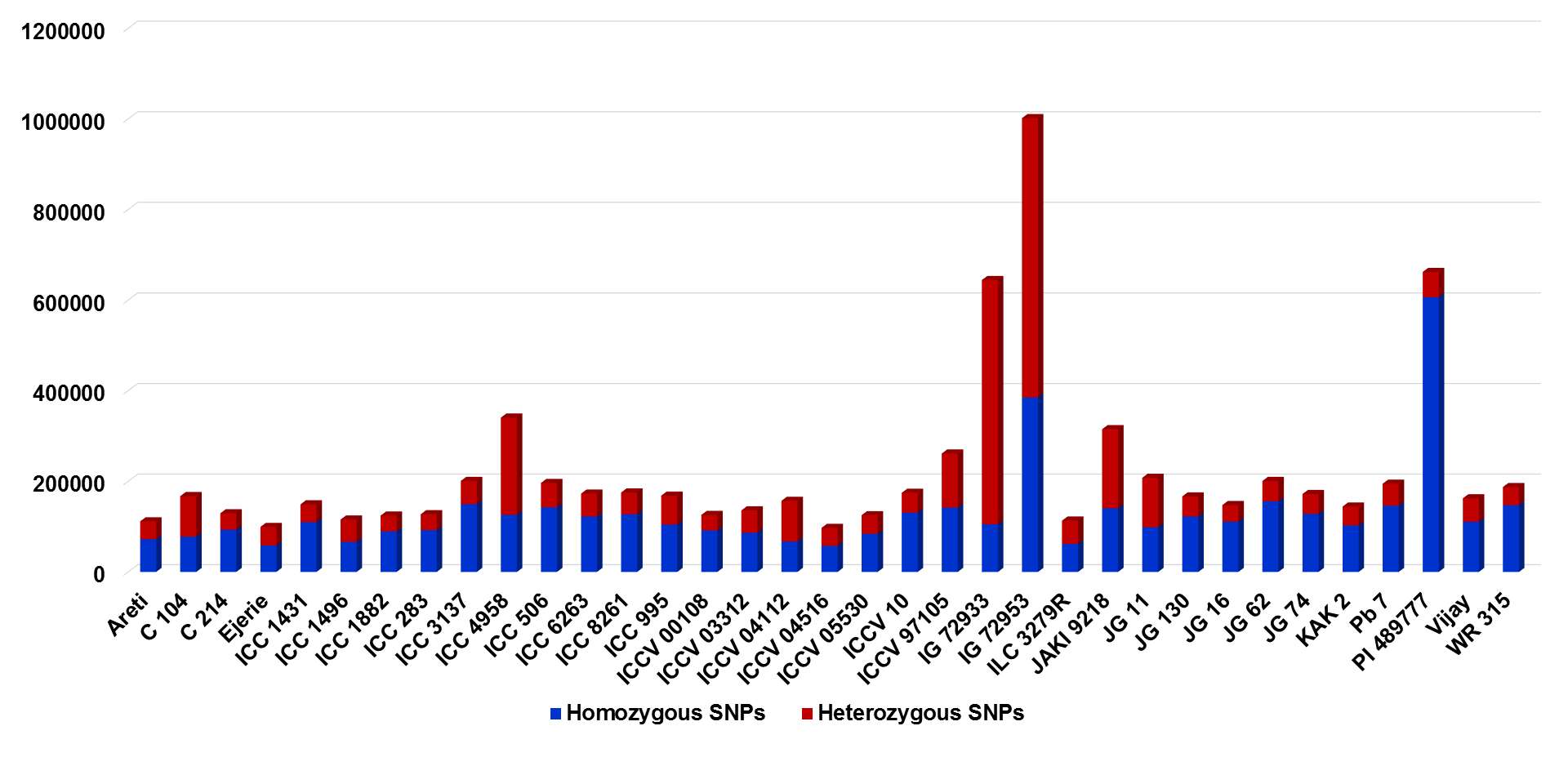

Supplement: Additional file 5: — Homozygous and heterozygous SNPs identified in each chickpea genotype used in the study. Maximum heterozugous SNPs are evident in IG 72933 while homozygous SNPs in PI 489777. (TIF 309 kb) [file 12870_2015_690_MOESM5_ESM.tif]
