# Supplementary material for: Whole genome re-sequencing reveals genome-wide variations among parental lines of 16 mapping populations in chickpea (Cicer arietinum L.)
Source: BMC Plant Biol. 2016 Jan 27;16(Suppl 1):10. doi: 10.1186/s12870-015-0690-3 (PMC4895712; doi:10.1186/s12870-015-0690-3)
Supplement: Additional file 21: — Summary of effects for the insertions. (DOCX 15 kb) [file 12870_2015_690_MOESM21_ESM.docx]

**Additional file 21: Summary of effects for insertions**

|  |  |  |  |  |  |  |  |  |
| --- | --- | --- | --- | --- | --- | --- | --- | --- |
| **Genotype** | **No of codon change and codon insertion** | **No of codon insertion** | **No of frame shift** | **No of intergenic insertions** | **No of introns affected** | **No of stop gained** | **NA** | **Insertions affecting genic regions (%)** |
| **Arerti** | 18 | 29 | 51 | 6,011 | 1,084 | 2 | 51 | 16.34 |
| **C 104** | 30 | 56 | 76 | 9,527 | 1,777 | 5 | 59 | 16.86 |
| **C 214** | 25 | 34 | 58 | 6,936 | 1,286 | 2 | 37 | 16.77 |
| **Ejerie** | 19 | 23 | 54 | 5,016 | 971 | 7 | 51 | 17.49 |
| **ICC 1431** | 25 | 42 | 65 | 8,012 | 1,517 | 4 | 41 | 17.03 |
| **ICC 1496** | 18 | 30 | 45 | 6,299 | 1,121 | 3 | 45 | 16.10 |
| **ICC 1882** | 20 | 25 | 50 | 6,632 | 1,223 | 5 | 45 | 16.54 |
| **ICC 283** | 23 | 31 | 51 | 6,751 | 1,266 | 5 | 38 | 16.85 |
| **ICC 3137** | 39 | 56 | 88 | 12,199 | 1,939 | 7 | 72 | 14.78 |
| **ICC 4958** | 67 | 95 | 136 | 24,330 | 3,680 | 8 | 113 | 14.02 |
| **ICC 506** | 34 | 54 | 85 | 11,708 | 1,893 | 7 | 78 | 14.96 |
| **ICC 6263** | 22 | 36 | 64 | 9,802 | 1,380 | 2 | 80 | 13.21 |
| **ICC 8261** | 25 | 45 | 73 | 9,913 | 1,550 | 2 | 66 | 14.52 |
| **ICC 995** | 22 | 32 | 61 | 9,124 | 1,438 | 10 | 68 | 14.53 |
| **ICCV 00108** | 17 | 22 | 34 | 7,767 | 1,016 | 1 | 50 | 12.24 |
| **ICCV 03312** | 14 | 35 | 54 | 7,325 | 1,179 | 3 | 53 | 14.83 |
| **ICCV 04112** | 22 | 28 | 54 | 9,165 | 1,587 | 5 | 50 | 15.54 |
| **ICCV 04516** | 17 | 20 | 39 | 5,023 | 842 | 2 | 44 | 15.37 |
| **ICCV 05530** | 23 | 29 | 51 | 6,444 | 1,167 | 4 | 45 | 16.41 |
| **ICCV 10** | 25 | 36 | 46 | 11,759 | 1,602 | 1 | 65 | 12.63 |
| **ICCV 97105** | 50 | 76 | 96 | 20,865 | 3,084 | 8 | 95 | 13.65 |
| **IG 72933** | 85 | 140 | 124 | 35,030 | 7,027 | 7 | 122 | 17.36 |
| **IG 72953** | 127 | 257 | 146 | 44,093 | 11,598 | 15 | 131 | 21.54 |
| **ILC 3279R** | 19 | 36 | 74 | 5,658 | 1,041 | 6 | 55 | 17.07 |
| **JAKI 9218** | 55 | 90 | 115 | 27,260 | 3,670 | 4 | 145 | 12.55 |
| **JG 11** | 23 | 34 | 50 | 15,350 | 2,100 | 2 | 81 | 12.52 |
| **JG 130** | 23 | 45 | 54 | 10,598 | 1,559 | 1 | 62 | 13.63 |
| **JG 16** | 25 | 43 | 49 | 8,535 | 1,400 | 1 | 41 | 15.04 |
| **JG 62** | 39 | 68 | 90 | 15,353 | 2,222 | 1 | 82 | 13.55 |
| **JG 74** | 30 | 48 | 65 | 9,254 | 1,739 | 3 | 56 | 16.84 |
| **KAK 2** | 19 | 29 | 52 | 7,830 | 1,262 | 5 | 74 | 14.74 |
| **PI 489777** | 81 | 157 | 135 | 36,596 | 8,774 | 7 | 107 | 19.96 |
| **Pb 7** | 29 | 54 | 79 | 11,940 | 1,826 | 4 | 60 | 14.24 |
| **Vijay** | 31 | 41 | 73 | 8,567 | 1,561 | 7 | 58 | 16.57 |
| **WR 315** | 30 | 60 | 71 | 13,413 | 1,990 | 1 | 75 | 13.76 |
