# Supplementary material for: Whole genome re-sequencing reveals genome-wide variations among parental lines of 16 mapping populations in chickpea (Cicer arietinum L.)
Source: BMC Plant Biol. 2016 Jan 27;16(Suppl 1):10. doi: 10.1186/s12870-015-0690-3 (PMC4895712; doi:10.1186/s12870-015-0690-3)
Supplement: Additional file 22: — Summay of effects for the deletions. (DOCX 15 kb) [file 12870_2015_690_MOESM22_ESM.docx]

| **Additional File 22: Summary of effects for the deletions** | | | | | | | |  |
| --- | --- | --- | --- | --- | --- | --- | --- | --- |
|  |  |  |  |  |  |  |  |  |
| **Genotype** | **No of codon change and codon deletion** | **No of codon deletion** | **No of frame shift** | **No of intergenic** | **No of intron** | **No of stop lost** | **NA** | **Deletions in coding regions (%)** |
| **Arerti** | 17 | 36 | 29 | 6,743 | 1,259 | 0 | 46 | 16.49 |
| **C 104** | 25 | 50 | 43 | 10,274 | 2,029 | 1 | 70 | 17.20 |
| **C 214** | 14 | 32 | 38 | 7,471 | 1,407 | 1 | 40 | 16.57 |
| **Ejerie** | 18 | 31 | 32 | 5,920 | 1,171 | 1 | 49 | 17.35 |
| **ICC 1431** | 26 | 34 | 38 | 8,782 | 1,787 | 1 | 44 | 17.61 |
| **ICC 1496** | 17 | 31 | 31 | 6,795 | 1,197 | 0 | 42 | 15.73 |
| **ICC 1882** | 19 | 25 | 27 | 7,365 | 1,409 | 0 | 43 | 16.65 |
| **ICC 283** | 24 | 32 | 31 | 7,346 | 1,428 | 1 | 46 | 17.02 |
| **ICC 3137** | 32 | 61 | 55 | 14,149 | 2,383 | 1 | 78 | 15.11 |
| **ICC 4958** | 47 | 101 | 81 | 25,572 | 4,061 | 1 | 113 | 14.31 |
| **ICC 506** | 25 | 54 | 49 | 13,744 | 2,257 | 1 | 90 | 14.71 |
| **ICC 6263** | 28 | 59 | 41 | 11,878 | 1,827 | 0 | 78 | 14.05 |
| **ICC 8261** | 27 | 53 | 36 | 11,879 | 1,998 | 0 | 82 | 15.02 |
| **ICC 995** | 32 | 50 | 33 | 10,841 | 1,832 | 0 | 75 | 15.14 |
| **ICCV 00108** | 13 | 24 | 20 | 7,949 | 1,153 | 0 | 58 | 13.13 |
| **ICCV 03312** | 25 | 38 | 31 | 8,530 | 1,437 | 0 | 46 | 15.15 |
| **ICCV 04112** | 34 | 36 | 42 | 10,300 | 1,929 | 0 | 61 | 16.46 |
| **ICCV 04516** | 15 | 20 | 21 | 6,025 | 1,036 | 0 | 42 | 15.25 |
| **ICCV 05530** | 24 | 49 | 27 | 7,480 | 1,391 | 0 | 51 | 16.53 |
| **ICCV 10** | 17 | 25 | 36 | 12,182 | 1,823 | 1 | 53 | 13.45 |
| **ICCV 97105** | 51 | 71 | 63 | 20,527 | 3,226 | 1 | 77 | 14.21 |
| **IG 72933** | 84 | 148 | 94 | 37,661 | 7,479 | 1 | 104 | 17.13 |
| **IG 72953** | 122 | 240 | 123 | 46,537 | 12,034 | 2 | 113 | 21.16 |
| **ILC 3279R** | 25 | 35 | 34 | 7,095 | 1,251 | 0 | 51 | 15.84 |
| **JAKI 9218** | 38 | 79 | 70 | 26,300 | 3,723 | 1 | 109 | 12.90 |
| **JG 11** | 23 | 32 | 34 | 15,286 | 2,243 | 0 | 72 | 13.18 |
| **JG 130** | 22 | 31 | 39 | 10,998 | 1,791 | 1 | 54 | 14.56 |
| **JG 16** | 16 | 30 | 30 | 8,912 | 1,541 | 1 | 49 | 15.29 |
| **JG 62** | 29 | 48 | 56 | 15,391 | 2,259 | 1 | 74 | 13.40 |
| **JG 74** | 31 | 56 | 44 | 10,378 | 1,972 | 0 | 59 | 16.77 |
| **KAK 2** | 22 | 42 | 38 | 9,105 | 1,530 | 0 | 60 | 15.12 |
| **PI 489777** | 109 | 199 | 95 | 38,502 | 9,232 | 1 | 87 | 19.98 |
| **Pb 7** | 20 | 46 | 47 | 13,548 | 2,109 | 1 | 63 | 14.04 |
| **Vijay** | 25 | 54 | 48 | 10,015 | 1,881 | 0 | 71 | 16.60 |
| **WR 315** | 23 | 37 | 42 | 13,276 | 2,066 | 1 | 57 | 13.99 |
